# Methamphetamine Increases Tubulo-Vesicular Areas While Dissipating Proteins from Vesicles Involved in Cell Clearance

**DOI:** 10.3390/ijms25179601

**Published:** 2024-09-04

**Authors:** Gloria Lazzeri, Paola Lenzi, Carla L. Busceti, Stefano Puglisi-Allegra, Michela Ferrucci, Francesco Fornai

**Affiliations:** 1 Human Anatomy, Department of Translational Research and New Technologies in Medicine and Surgery, University of Pisa, 56126 Pisa, Italy; gloria.lazzeri@unipi.it (G.L.); paola.lenzi@unipi.it (P.L.); 2 IRCCS—Istituto di Ricovero e Cura a Carattere Scientifico, Neuromed, 86077 Pozzili, Italy; carla.busceti@neuromed.it (C.L.B.); stefano.puglisiallegra@neuromed.it (S.P.-A.)

**Keywords:** Parkinson’s disease, neurodegeneration, drugs of abuse, ultrastructural pathology, catecholaminergic degeneration, autophagolysosomes, mitochondria

## Abstract

Cytopathology induced by methamphetamine (METH) is reminiscent of degenerative disorders such as Parkinson’s disease, and it is characterized by membrane organelles arranged in tubulo-vesicular structures. These areas, appearing as clusters of vesicles, have never been defined concerning the presence of specific organelles. Therefore, the present study aimed to identify the relative and absolute area of specific membrane-bound organelles following a moderate dose (100 µM) of METH administered to catecholamine-containing PC12 cells. Organelles and antigens were detected by immunofluorescence, and they were further quantified by plain electron microscopy and in situ stoichiometry. This analysis indicated an increase in autophagosomes and damaged mitochondria along with a decrease in lysosomes and healthy mitochondria. Following METH, a severe dissipation of hallmark proteins from their own vesicles was measured. In fact, the amounts of LC3 and p62 were reduced within autophagy vacuoles compared with the whole cytosol. Similarly, LAMP1 and Cathepsin-D within lysosomes were reduced. These findings suggest a loss of compartmentalization and confirm a decrease in the competence of cell clearing organelles during catecholamine degeneration. Such cell entropy is consistent with a loss of energy stores, which routinely govern appropriate subcellular compartmentalization.

## 1. Introduction

When analyzing cytopathology induced by methamphetamine (METH) and occurring in specific degenerative disorders, such as Parkinson’s disease (PD), a constant failure of vesicular compartments commonly called the autophagolysosome system is evident [1,2,3,4,5,6,7]. In fact, a deficiency in autophagolysosome activity induces degeneration of catecholamine neurons [8,9,10] with massive clusterization of abnormal vesicles. This occurs ex vivo within dopamine (DA)-containing substantia nigra cells from PD patients [11] and can be replicated in vitro. In fact, this can be reproduced in DA-containing cells by administration of abnormal amounts of alpha-synuclein [5] or administration of autophagy inhibitors [10], or following METH administration [12]. In line with this, recent evidence, confirming previous findings, indicates that METH, in a similar manner to PD, produces a cytopathology where aggregates of p62 and polyubiquitin co-localize within cell spots along with vesicular membrane organelles [12,13,14]. In this way, accumulation of various vesicular organelles such as autophagosomes contributes to polymorphic aggregates, which characterize the cytopathology of neurodegeneration both in PD [11] and following METH exposure [12]. Although subcellular evidence suggests a conspicuous number of organelles [11,12,13], no study to date has quantified these vesicular areas in the course of neurodegeneration compared with baseline conditions. In addition, no marker has been used to better assess the specific nature of these vesicles and the relative abundance of each specific kind of vesicle. Finally, the potential clustering or dissipation of marker proteins within/from this vacuolar compartment has never been assessed through in situ stoichiometry.

Therefore, in the present study, the following points were investigated: (i) The amount of vacuolar area within the cytosol during METH-induced degeneration was compared with that occurring during baseline conditions. (ii) The relative abundance of specific vacuoles was assessed following immunostaining against specific protein markers on both light and electron microscopy. (iii) The quantities of proteins occurring within subcellular vesicles or scattered within the cytosol were measured following METH administration compared with baseline conditions. This was carried out by in situ immunogold stoichiometry. In this way, the authentic amount, compartmentalization and/or dissipation of competent molecules either distributed in the whole cytosol or allocated within specific vesicles was measured in situ. In detail, the amount and placement of LC3 were detected in relation to the autophagosome compartment. The distribution of p62 was assessed to analyze the shuttling of polyubiquitinated substrates within autophagosomes. The distribution of P20S was measured to analyze the compartmentalization of the proteasome within autophagosomes to produce autophagoproteasomes. The distribution of LAMP1 was measured to mark lysosomes. The quantity of vacuolar Cathepsin-D (Cat-D) was assessed as a typical lysosomal enzyme. The quantities of healthy and damaged mitochondria were directly measured within catecholamine cells under baseline conditions and following METH administration.

Each molecule was assessed alone and in combination, both in the whole cytosol and within specific compartments. Dissipation or clustering of these specific proteins within vacuoles is fundamental to enable cell clearance by removing molecular cargoes and altered mitochondria. A dose of 100 µM METH was selected based on our previous dose–response studies [12,15]. This dose produces moderate cell loss, where spared cells are available to assess the related cytopathology. In fact, when cell loss is moderate, most of the cells (63% as counted following H&E) are able to survive and may develop a fine cytopathology, which makes it possible to dissect crucial subcellular areas involved in the molecular mechanisms of catecholamine-induced cell damage.

## 2. Results

### 2.1. A Moderate Dose of METH (100 µM) Produces Partial Cell Loss and Cell Degeneration

**Figure 1:**
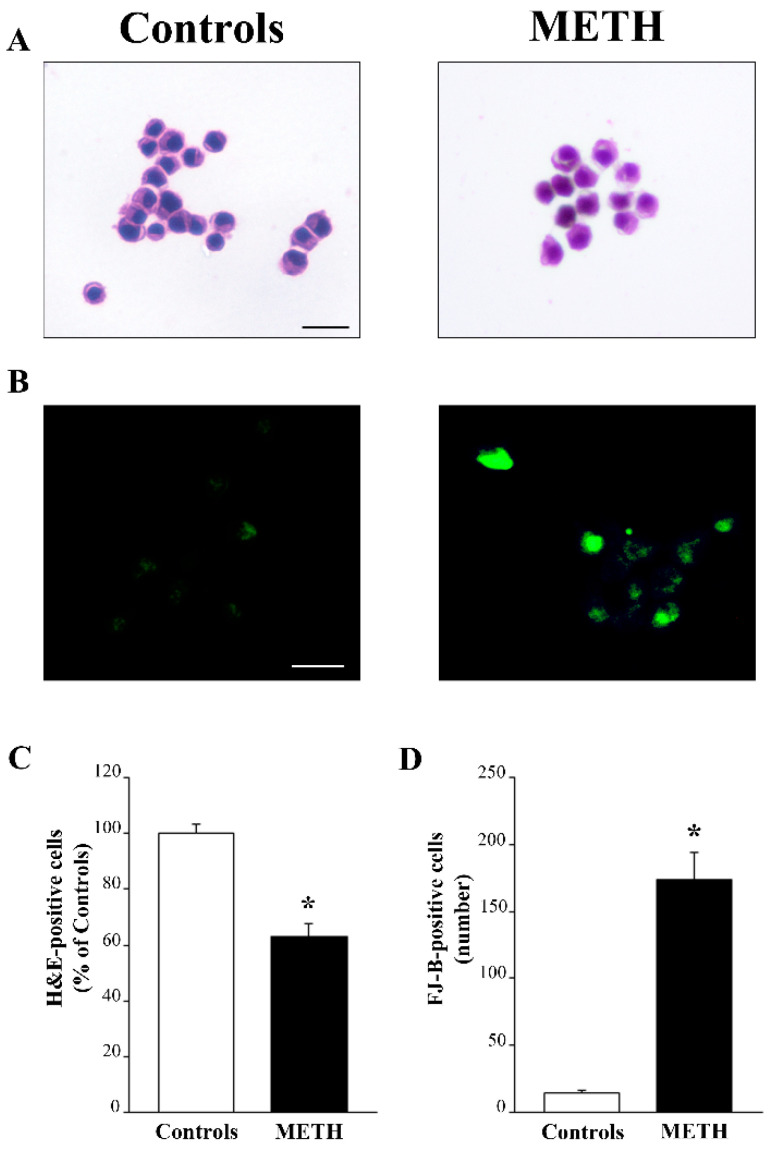
In PC12 cells, a moderate dose of METH (100 µM) induces moderate cell death and noticeable cell damage. (**A**) Representative pictures of H&E-stained cells from controls and METH-treated cells, respectively. Apart from decreased cell number (63% of controls), there is evident cell pathology induced by METH, with pale vacuolar cytosolic areas, irregular shape and smaller size compared with the homogenously stained cytosol and regular cell shape of the control. (**B**) FJ-B histofluorescence from controls and METH-treated cells, respectively, where degenerating cells are evident mostly among METH-treated cells. (**C**) The graph reports the percentage of H&E-stained viable cells following METH compared with control conditions according to the criteria expressed in the Methods, extended statistics. (**D**) The graph reports that FJ-B-positive cells (absolute number), occur in excess (almost twelvefold) in METH-treated cells compared with controls. Values in C are given as the mean percentage of controls (where controls = 100%) ± S.E.M. Values in D are given as the mean ± S.E.M. of controls and METH-treated cells, respectively. Comparisons between groups were carried out through one-way ANOVA with Scheffe’s post hoc analysis. The null hypothesis was rejected for *p* < 0.05. * *p* < 0.05 compared with controls. Scale bars (**A**,**B**) = 15 µm.

Administration of METH (100 µM) produces cell loss that is slight (37% of cell loss compared with controls following H&E). This implies that 63% of the cells are able to survive and may develop crucial subcellular domains where the ultrastructural pathology induced by METH can be described. The effects of METH (100 µM) on cell integrity were assessed following H&E histochemistry as shown in representative Figure 1A and following FJ-B histofluorescence as shown in representative Figure 1B. This is reported in the graphs of Figure 1, where cell loss following H&E is expressed as the mean percentage of controls (graph in Figure 1C), while cell degeneration following FJ-B is reported as the mean number of fluorescent cells (graph in Figure 1D). Both histochemical procedures converge in documenting a moderate amount of cell death, which validates previous studies carried out using a wide range of dose–response curves as reported in the extended statistics [12,15]. These studies already documented moderate (up to roughly 40%) cell loss following this dose of METH when analyzed through H&E histochemistry. In these experimental conditions, the amount of cell damage (as assessed by FJ-B fluorescence) involves most of the cells. These experimental conditions are ideal to assess cytopathology affecting surviving cells; under these conditions, degenerative phenomena occur while still allowing cell survival.

### 2.2. Specific Vesicular Markers Increase Following METH (100 µM)

**Figure 2:**
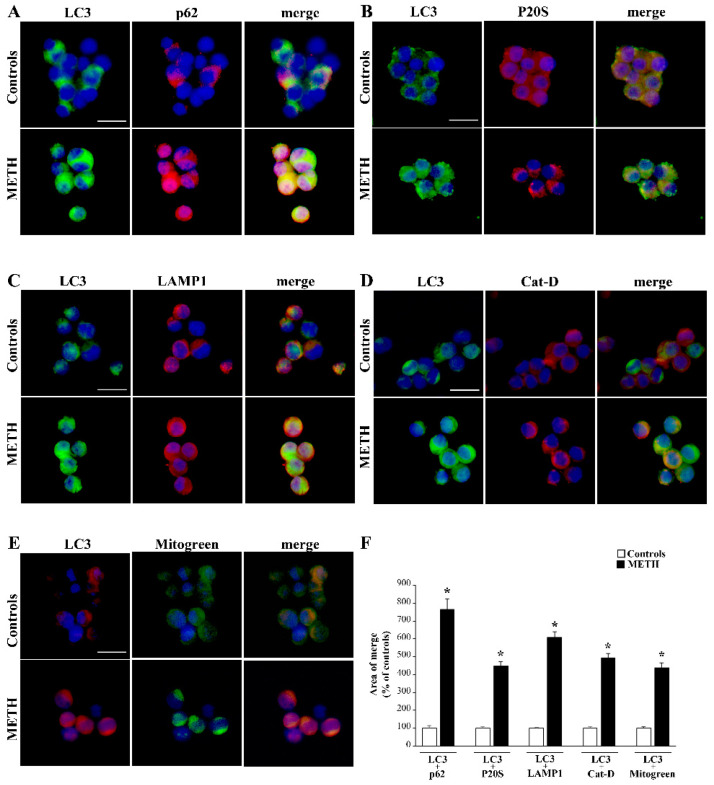
Specific vesicular markers and their overlap with LC3 increase following METH (100 µM). As shown in representative pictures in Figure 2, in PC12 cells, METH markedly increases the amount of LC3 compared with control conditions both alone and in combination with various antigens, which are also modified: (**A**) p62; (**B**) P20S; (**C**) LAMP-1; (**D**) Cat-D and (**E**) Mitogreen. All these antigens increase following METH (except p20S), and an increase is measured in their area of overlap with LC3 (including P20S). It is important to emphasize that Mitogreen is a non-specific mitochondrial marker, since it stains both healthy and damaged mitochondria. It is difficult to interpret the significance of this latter merging based solely on light microscopy. In fact, this could be due to either the placement of LC3 over the mitochondrial structure or the mitophagy of altered mitochondria within engulfed autophagosomes. Again, this might be due to disrupted mitochondrial structures co-localizing with LC3 outside of any specific compartment. (**F**) reports the graph showing the area of overlapping immunofluorescence between each antigen and LC3. As reported, all antigens produce a remarkable area of overlap with LC3 following METH (100 µM) compared with control conditions. Values are given as the mean percentage compared with controls, assuming controls = 100% ± S.E.M. Please refer also to Table 1, which reports the absolute overlapping area, expressed in µm^2^). Comparisons between groups were carried out through one-way ANOVA with Scheffe’s post hoc analysis. The null hypothesis was rejected for *p* < 0.05. * *p* < 0.05 compared with controls. Scale bars = 12 µm.

As shown in the representative pictures in Figure 2, METH markedly increases the amount of the autophagosome marker LC3 compared with control conditions (representative pictures in Figure 2A–E). In contrast, no noticeable increase was documented for the proteasome marker P20S (representative pictures in Figure 2B). Similarly, representative pictures show a remarkable increase in p62 (sequestosome) induced by METH (representative pictures in Figure 2A). Such an increase is also noticeable for the membrane lysosome marker LAMP1 (representative pictures in Figure 2C) and the lysosome enzyme Cat-D (representative pictures in Figure 2D).

Again, when mitochondria were stained using the non-specific marker MitoTracker Green (Mitogreen, Thermo-Fisher Scientific, Waltham, MA, USA), a remarkable increase in total mitochondria was evident following METH compared with controls (representative pictures in Figure 2E). In fact, Mitogreen is a non-specific mitochondrial marker since it stains both healthy and damaged mitochondria. The increased staining of these markers were confirmed when merging LC3 with p62 (representative picture in Figure 2A); LC3 and P20S (representative pictures in Figure 2B); LC3 and LAMP1 (representative pictures in Figure 2C) and LC3 with Cat-D (representative pictures in Figure 2D); LC3 and Mitogreen (representative pictures in Figure 2E).

Since all merging patterns included LC3 staining it is not surprising that even in the case of P20S we documented an increased merging due to the massive augmentation in LC3 and the steady amount of P20S, which co-localize with LC3. The increase in Mitogreen following METH was already reported [16]; in the present study it was merged for the first time with the autophagosome marker LC3. It is difficult to interpret the significance of such a merging only based on light microscopy. In fact, this could be due either to the placement of LC3 over the mitochondrial structure, or the mitophagy of altered mitochondria within engulfed autophagosomes. Again, this might be due to disrupted mitochondrial structures co-localizing with LC3 out of any specific compartment. To solve this issue electron microscopy is required, as carried out in the second part of this study, to detail the amount and placement concerning such an increase.

The graph in Figure 2F shows the area of merging immunofluorescence for each antigen with LC3. As reported, p62, P20S, LAMP1, Cat-D and Mitogreen show remarkable overlaps with LC3 following METH (100 µM) compared with controls (graph in Figure 2F, which reports the percentage compared with controls, and Table 1, which reports the actual overlapping area expressed in µm^2^). This overlap on light microscopy (evident in representative pictures and overlapping areas counted in the graph) for vacuolar proteins following METH administration is commonly interpreted as the result of METH-induced large membrane vesicles filled with antigens due to a reduced/impeded progression in the autophagoproteasome/lysosome pathway [8,9,17]. Indeed, the signal overlap of these antigens does not prove their concomitant placement within autophagoproteasomes/lysosomes. In fact, as hypothesized by Rami [18,19,20] and confirmed by Lazzeri et al. [9] in line with Mastroiacovo [21,22], merging of immunofluorescence following METH or even brain ischemia does not reveal much about which cell compartment accumulates the antigens. In fact, Lazzeri et al. [9] demonstrated that, following METH, increased quantities of LC3 fluorescent spots occur outside of the autophagoproteasome/lysosome compartment when analyzed on TEM.

Thus, even when considering increased immunofluorescent spots for other antigens, the inference about their potential placement within specific compartments needs to be validated by TEM. Thus, the present data on light microscopy should be simply interpreted as increased quantities of single or merged antigens that, in baseline conditions, are mostly stored within the autophagolysosome system. The actual placement in the cell following METH remains unsolved by data obtained by immunofluorescence even when analyzed by confocal microscopy. Again, this calls for a finer analysis using TEM and immunogold.

### 2.3. TEM Shows Variations of Tubulo-Vesicular Structures Following METH

**Figure 3:**
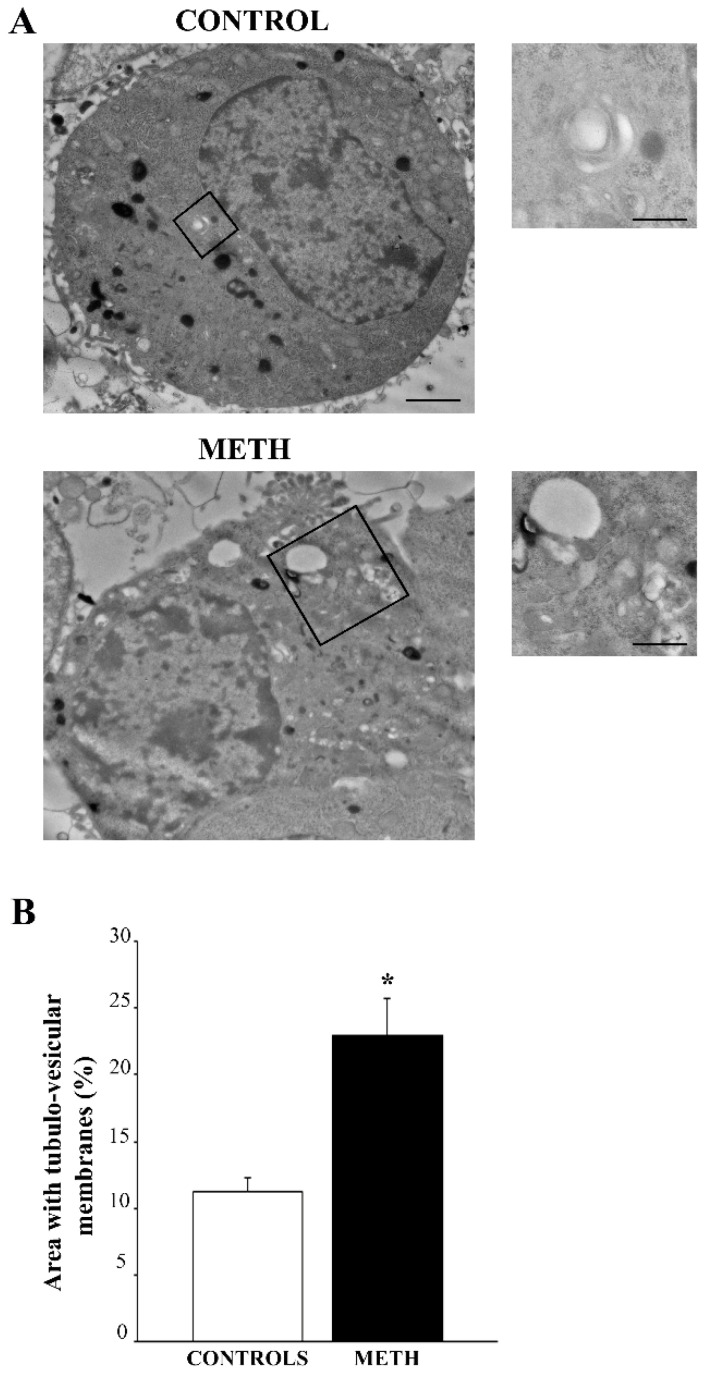
METH alters tubulo-vesicular domains in PC12 cells on TEM. (**A**) Representative pictures from control (upper lane)- and METH (lower lane)-treated PC12 cells. In each of these pictures, the inset provides a higher magnification. (**B**) The graph reports the area covered by tubulo-vesicular structures, which is expressed as the mean percentage ± S.E.M. of the area of the whole cytosol (n = 30). Comparisons between groups were carried out through one-way ANOVA with Scheffe’s post hoc analysis. The null hypothesis was rejected for *p* < 0.05. * *p* < 0.05 compared with controls. Picture scale bars = 689 nm. Inset scale bars = 229 nm (Control); 300 nm (METH).

When plain electron microscopy was carried out within METH-treated cells, a classic sub-cellular pathology was evident, which confirms that recently reported by Ferrucci et al. (2024) [12]. In this study, light and electron microscopy were carried out in combination following METH to assess the matching between these procedures in detailing METH-induced cytopathology. The increase in METH-induced tubulo-vesicular structures is confirmed in the present study as showed in representative Figure 3A. In fact, when the area of tubulo-vesicular structures is measured this takes over 20% of the whole cytosol. This corresponds to an increase exceeding 200% of controls (graph in Figure 3B). It is remarkable that METH produces a general increase in tubulo-vesicular structures in the whole cytosol and markedly clusters these vesicles within discrete cytosolic domains occupying a rough area of 2 µm^2^. In fact, these tubulo-vesicular domains are 4.5-fold more abundant in the cytosol of METH-treated cells than in the cytosol from control cells. Thus, following METH, tubulo-vesicular areas increase according to a pattern of clusters, consisting of vesicles packed within an area averaging 2 µm^2^.

This is consistent with the domains of cytopathology described in the course of METH-induced toxicity [12]. Tubulo-vesicular areas may be further analyzed through immunocytochemistry joined with plain microscopy concerning the occurrence of specific organelles as reported in percentage within Table 2. The counts of recognizable structures based on immunogold staining joined with plain ultrastructural morphology indicates mostly autophagosomes. In fact, autophagosome-like organelles cover most of the domain area being scanned (for both the METH and control groups). Indeed, as shown in Table 2, the relative quantities of most organelles (provided as percentage of the area) are modified in METH-treated cells compared with controls. The rough data for mean areas used to calculate the percentage of Table 2 are reported here for each organelle. For instance, autophagosome area is significantly increased following METH (0.542 ± 0.021 µm^2^) compared with control conditions (0.447 ± 0.026 µm^2^). Lysosomes cover an area of 0.084 ± 0.007 µm^2^ and 0.373 ± 0.022 µm^2^ (for the METH and control groups, respectively), while damaged mitochondria occupy an area of 0.284 ± 0.020 µm^2^ and 0.128 ± 0.010 µm^2^ (for the METH and control groups, respectively). In contrast, healthy mitochondria cover an area of 0.081 ± 0.006 µm^2^ and 0.217 ± 0.010 µm^2^ (for the METH and control groups, respectively). Multivesicular bodies (MVBs) take an area of 0.100 ± 0.006 µm^2^ and 0.107 ± 0.005 µm^2^ (for the METH and control groups, respectively). Within tubulo-vesicular cytosolic domains, a large part of the vesicular content is difficult to recognize through immunogold, and it is likely to represent endosomes and/or retromers, along with a number of structures derived from the trans-Golgi network (TGN). These unidentified tubulo-vesicular structures are in excess following METH compared with control conditions in the whole cytosol and, most intensely, within the 2 µm^2^ clusters. In detail, in the whole cytosol, tubulo-vesicular areas are in great excess (more than 200%) following METH compared with control conditions (Figure 3). When considering the quantity of 2 µm^2^ wide clustered tubulo-vesicular domains, METH increases the number 4.5-fold compared to that of controls. Within the cluster domain, the quantity of non-deciphered vesicles is greatly increased by METH both in percentage (Table 2) and in absolute area (0.909 ± 0.034 µm^2^ and 0.728 ± 0.036 µm^2^ for the METH and control groups, respectively). This is in line with an increase in unstained atypical vesicles produced by METH [9], and it is likely to depend on the dissipation of hallmark proteins from proper vesicles, which is caused by METH, as will be analyzed in the following paragraphs.

### 2.4. METH Modifies the Amount and Placement of Specific Hallmark Proteins in PC12 Cells as Assessed by Immunogold

#### 2.4.1. LC3

**Figure 4:**
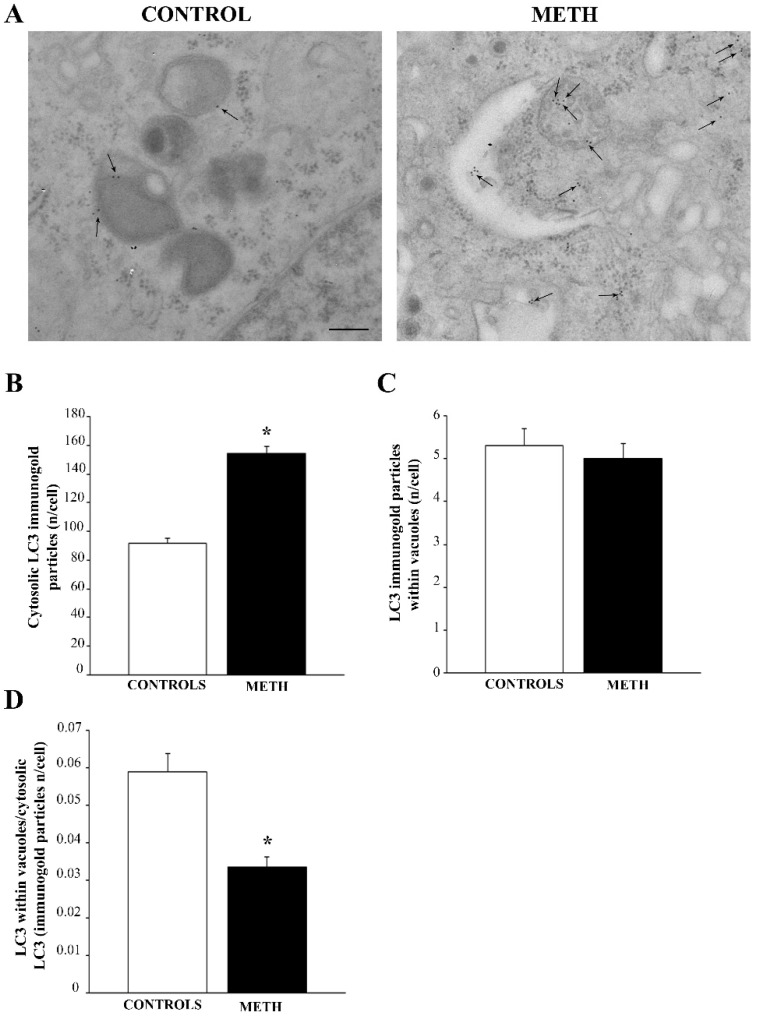
In PC12 cells, METH increases LC3 immunogold in the whole cytosol and dissipates LC3 from vacuoles. (**A**) Representative pictures showing Control and METH, respectively. Arrows indicate LC3 immunogold. METH markedly increases LC3 in the whole cytosol, although the vacuolar amount of LC3 does not change. (**B**) A graph reporting the number of LC3 particles in the whole cytosol. (**C**) A graph reporting the amount of LC3 within vacuoles. (**D**) A graph reporting the decrease in the ratio of vacuolar to cytosolic LC3 produced by METH. This indicates that, although METH increases LC3 in the whole cell, this autophagosome-preferring protein is dissipated from autophagosome vacuoles towards the non-particulate cytosol. Values are expressed as the mean + S.E.M. of n = 30 cells per group. Comparisons between groups were carried out through one-way ANOVA with Scheffe’s post hoc analysis. The null hypothesis was rejected for *p* < 0.05. * *p* < 0.05 compared with controls. Scale bar (**A**): 125 nm.

The amount of immunogold staining for LC3 particles was markedly increased when the whole cytosol was considered (representative pictures in Figure 4A and graph Figure 4B), however the vacuolar amount of LC3 was not significantly changed following METH exposure (Figure 4C). This led to a marked decrease in the ratio between vacuolar and cytosolic LC3 (Figure 4D). This indicates that, although METH increases LC3 in the whole cell, this autophagosome-preferring protein is dissipated from autophagosome vacuoles towards the non-particulate cytosol. Such a dissipation of LC3 from vacuoles indicates a loss of compartmentalization and a decrease in competent autophagy vacuoles, confirming previous reports, where LC3 dissipation was measured in brain ischemia (Mastroiacovo et al., 2022 [22]).

#### 2.4.2. p62

**Figure 5:**
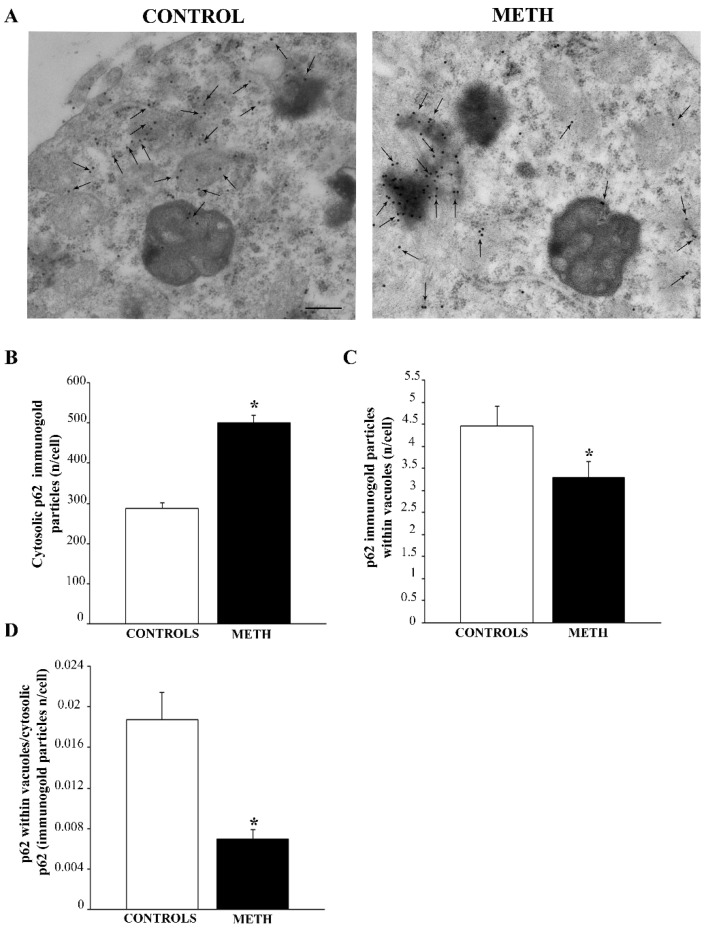
In PC12 cells, METH increases p62 immunogold in the whole cytosol, while decreases p62 from vacuoles. (**A**) Representative pictures showing Control and METH, respectively. Arrows indicate p62 immunogold. The amount markedly increases in the whole cytosol although vacuolar amount of p62 was reduced following METH exposure. (**B**) A graph reporting the number of p62-stained immunogold particles in the whole cytosol. (**C**) A graph reporting the amount of p62 within vacuoles. (**D**) A graph reporting the decrease in the ratio of vacuolar to cytosolic p62 produced by METH. This indicates that, although METH increases p62 in the whole cell, this autophagosome-preferring protein is dissipated from autophagosome vacuoles towards the non-particulate peri-vacuolar cytosol. Values are expressed as the mean + S.E.M. of n = 30 cells per group. Comparisons between groups were carried out through one-way ANOVA with Scheffe’s post hoc analysis. The null hypothesis was rejected for *p* < 0.05. * *p* < 0.05 compared with controls. Scale bar (**A**): 172 nm.

As with LC3, the sequestosome protein p62 was increased following METH compared with controls (representative pictures in Figure 5A and graph in Figure 5B). However, most p62 was counted in close proximity of vesicular organelles instead of being compartmentalized within vacuoles (representative pictures in Figure 5A and graph in Figure 5C). This explains why the ratio provided by the quantity of p62 immunogold within vacuoles with respect to total cytosol p62 is markedly decreased following METH (graph in Figure 5D). Again, this indicates a dissipation of p62 from vacuoles towards the peri-vacuolar cytosol. In turn, this suggests that the physiological role of p62 in shuttling polyubiquitinated substrates from cytosol to autophagosomes [23,24] is impeded by METH administration.

#### 2.4.3. P20S

**Figure 6:**
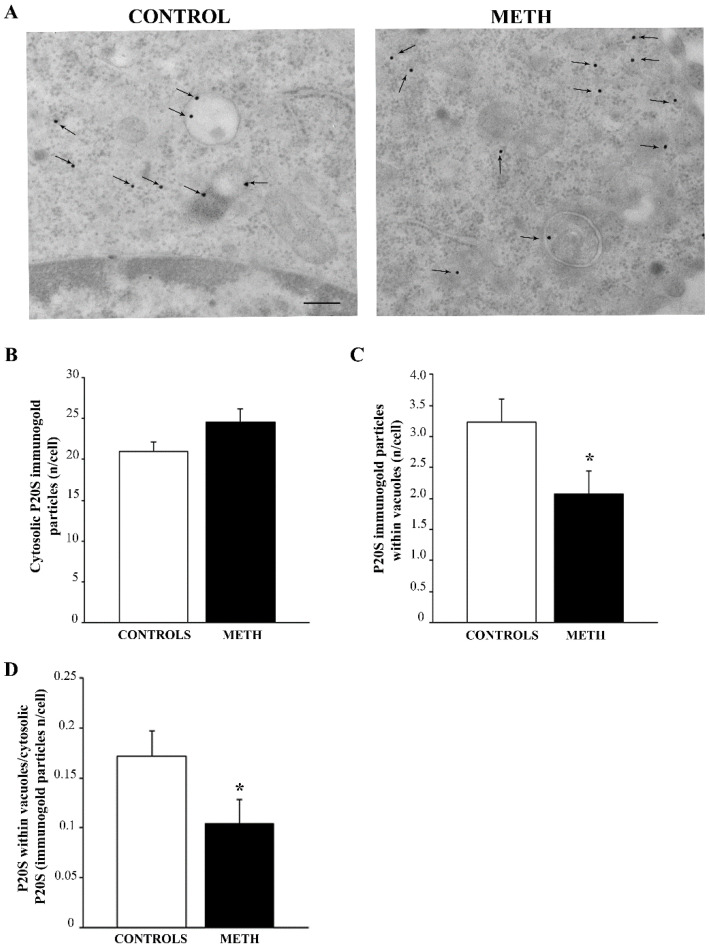
In PC12 cells, METH does not alter cytosolic P20S despite reducing vacuolar P20S. (**A**) Representative pictures showing Control and METH, respectively. Arrows indicate P20S immunogold. The amount varies following METH when counted in the whole cytosol, although vacuolar amount of p20S is following METH exposure. (**B**) A graph reporting the number of P20S-stained immunogold particles in the whole cytosol. (**C**) A graph reporting the amount of P20S within vacuoles. (**D**) A graph reporting the decrease in the ratio of vacuolar to cytosolic P20S produced by METH. This indicates that, despite METH does not alter the amount of P20S in the whole cytosol, this proteasome-preferring protein is dissipated from autophagoproteasome vacuoles [16] towards the non-particulate peri-vacuolar cytosol. Values are expressed as the mean + S.E.M. of n = 30 cells per group. Comparisons between groups were carried out through one-way ANOVA with Scheffe’s post hoc analysis. The null hypothesis was rejected for *p* < 0.05. * *p* < 0.05 compared with controls. Scale bar (**A**): 172 nm.

METH administration did not alter the amount of P20S in the whole cytosol compared with controls (representative pictures in Figure 6A and graph in Figure 6B). However, when the amount of P20S was counted within vacuoles a loss of P20S was noticed (graph in Figure 6C). Similarly, a marked decrease in the ratio between vacuolar and cytosolic P20S (graph in Figure 6D) was measured. This is in line with METH-induced impaired shuttling of P20S within autophagosome vesicles [9]. This is consistent with an impaired shuttling of P20S and polyubiquitinated substrates through p62 within autophagosomes [9,23,24] autophagosomes. This phenomenon is likely to be relevant for impairing the formation of autophagoproteasomes and the specific enzymatic activity, which occurs within this vacuolar compartment [25].

#### 2.4.4. LAMP1

**Figure 7:**
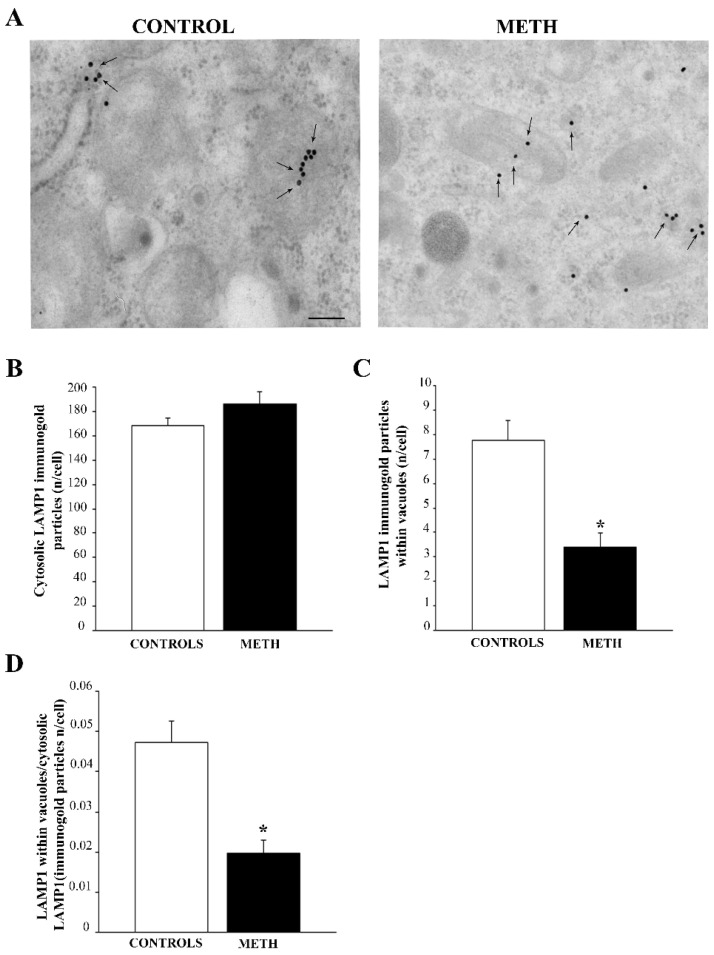
METH does not alter cytosolic LAMP1 despite reducing LAMP1 vacuolar compartmentalization in PC12 cells. (**A**) Representative pictures showing Control and METH, respectively. Arrows indicate LAMP1 immunogold. The quantity does not change following METH when counted in the whole cytosol, although the vacuolar amount of LAMP1 is markedly reduced following METH exposure. (**B**) A graph reporting the number of LAMP1-stained immunogold particles in the whole cytosol. (**C**) A graph reporting the amount of LAMP1 within vacuoles. (**D**) A graph reporting the decrease in the ratio of vacuolar to cytosolic LAMP1 produced by METH. This indicates that, although METH does not alter the amount of LAMP1 in the whole cytosol, this lysosome-preferring protein is dissipated from lysosome/autophagolysosome vacuoles towards the non-particulate peri-vacuolar cytosol. Values are expressed as the mean + S.E.M. of n = 30 cells per group. Comparisons between groups were carried out through one-way ANOVA with Scheffe’s post hoc analysis. The null hypothesis was rejected for *p* < 0.05. * *p* < 0.05 compared with controls. Scale bar (**A**): 125 nm.

When the total cytosolic quantity of LAMP1 is counted, this lysosome marker is not modified in the whole cytosol (representative pictures in Figure 7A and graph in Figure 7B), although its placement within vacuoles is reduced (representative pictures in Figure 7A and graph in Figure 7C). This was further evidenced by the ratio between vacuolar and cytosolic LAMP1 (graph in Figure 7D). The deficiency of lysosomal LAMP1 may provide an explanation for the reduced lysosomal activity in the context of autophagolysosome failure, which occurs following METH administration. Again, this explains why the subcellular analysis of tubulo-vesicular domains previously reported (Table 2) indicates a decrease in lysosomes following METH.

#### 2.4.5. Cat-D

**Figure 8:**
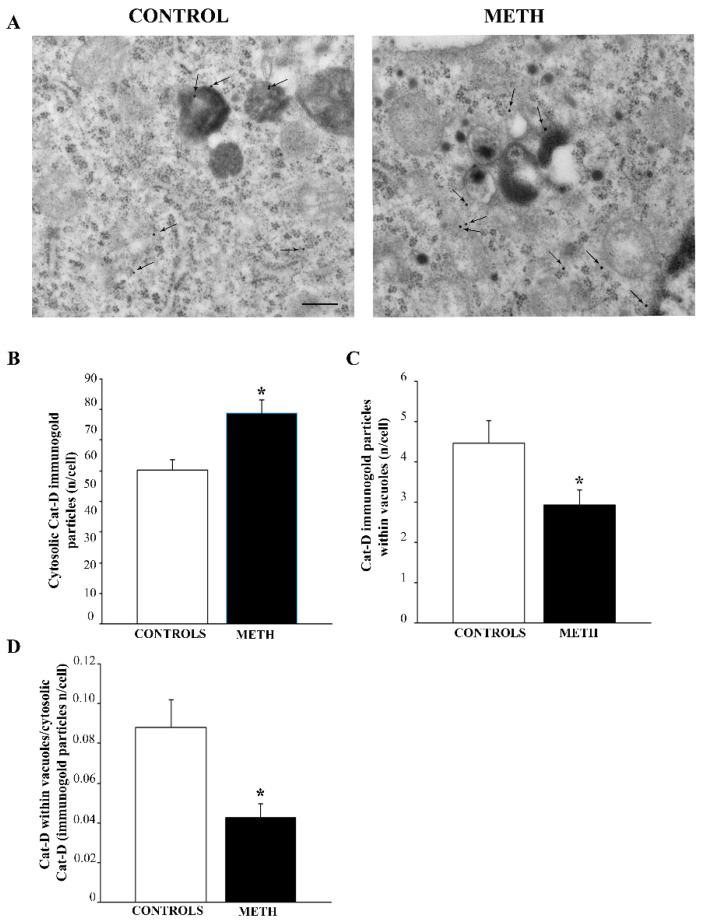
METH increases cytosolic Cat-D while reducing the amount of Cat-D in the vacuoles of PC12 cells. (**A**) Representative pictures showing Control and METH, respectively. Arrows indicate Cat-D immunogold. The amount strongly increases following METH when counted in the whole cytosol, although the vacuolar amount of Cat-D is markedly reduced following METH exposure. (**B**) A graph reporting the number of Cat-D-stained immunogold particles in the whole cytosol. (**C**) A graph reporting the amount of Cat-D within vacuoles. (**D**) A graph reporting the dramatic decrease in the ratio of vacuolar to cytosolic Cat-D produced by METH. This indicates that, METH severely decreases Cat-D compartmentalization both as a consequence of increased cytosolic Cat-D and reduced vacuolar Cat-D. Thus, this lysosome-preferring enzyme is profoundly dissipated from lysosome/autophagolysosome vacuoles towards the non-particulate peri-vacuolar cytosol. Values are expressed as the mean + S.E.M. of n = 30 cells per group. Comparisons between groups were carried out through one-way ANOVA with Scheffe’s post hoc analysis. The null hypothesis was rejected for *p* < 0.05. * *p* < 0.05 compared with controls. Scale bar (**A**): 172 nm.

METH produces a massive increase in the cytosolic amount of Cat-D (representative pictures in Figure 8A and graph in Figure 8B), while only a few vesicular amount of Cat-D are present (representative pictures in Figure 8A and graph in Figure 8C). This led to a marked decrease in the ratio between vacuolar and cytosolic Cat-D (graph in Figure 8D). This is consistent with a loss of lysosomal competence and a potential risk of inappropriate Cat-D-induced degradation within unusual cytosolic compartments.

#### 2.4.6. Combined Immunogold

**Figure 9:**
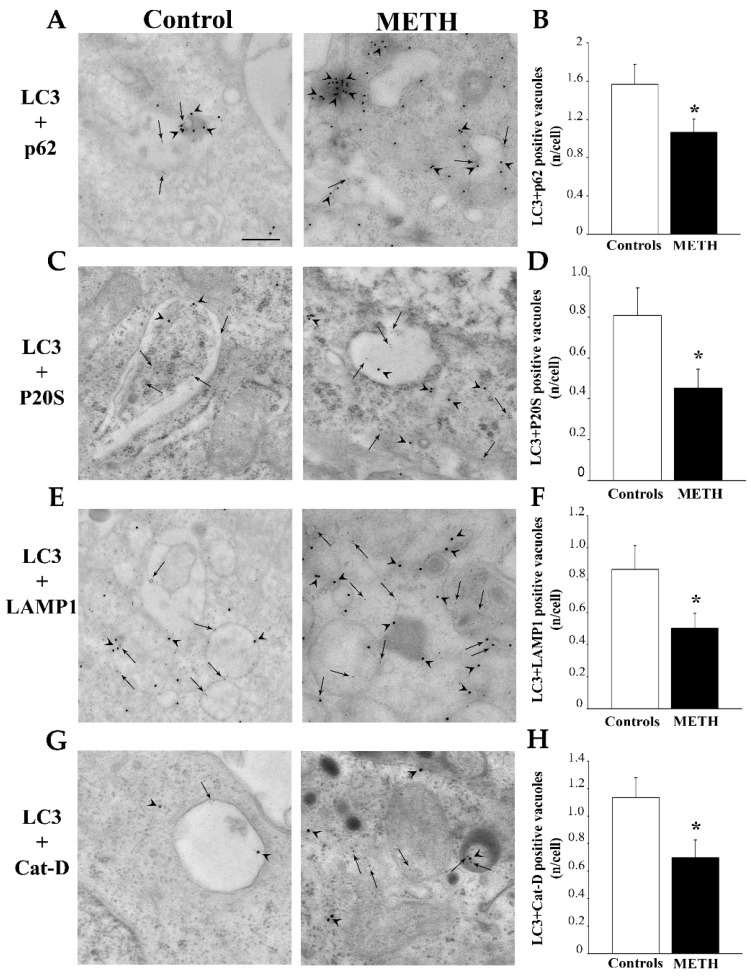
In PC12 cells METH dissipates all double immunogold keeping constant LC3 immunocytochemistry. When double immunogold was carried out, antibodies against LC3 (arrows) were challenged with all other antibodies (arrowheads). The representative pictures (**A**) and graph (**B**) report double staining of LC3 and p62, which indicate that METH produces a marked loss of LC3+p62 positive vacuoles. Representative pictures (**C**) and graph (**D**) show double immunogold for LC3+P20S, which provides similar results since METH decreases the vacuolar amount of both antigens. The representative pictures (**E**) and graph (**F**) report double immunogold of LC3+LAMP1, which indicates a severe loss of autophagolysosomes. This is likely to be due to impaired merging between autophagosomes and lysosomes. The representative pictures (**G**) and graph (**H**) report double immunogold of LC3+Cat-D. Again, the decrease is consistent with previous findings indicating a loss of autophagolysosomes. Values are expressed as the mean + S.E.M. of n = 30 cells per group. Comparisons between groups were carried out through one-way ANOVA with Scheffe’s post hoc analysis. The null hypothesis was rejected for *p* < 0.05. * *p* < 0.05 compared with controls. Scale bars: 125 nm.

When double immunogold was carried out, antibodies against LC3 were challenged with antibodies against other antigens as reported for light microscopy. Double staining of LC3 and p62 indicates that METH produces a marked loss of LC3+p62 positive vacuoles (representative pictures in Figure 9A and graph in Figure 9B). This is consistent with a dissipation of both LC3 and p62 (Figure 4 and Figure 5) from vacuoles compared with cytosol in close proximity of vacuoles reported above for single immunogold. These implies that merged immunofluorescent spots shown light microscopy should be interpreted as independent from vacuoles. In fact, these areas featuring both LC3 and p62 which are increased following METH on light microscopy (representative pictures in Figure 2A and graph in Figure 2F) and electron microscopy occur outside of vacuoles, within non-specific cell compartments (representative pictures in Figure 9A and graph in Figure 9B).

Merging of LC3+P20S produced similar results since METH decreased the vacuoles featuring both antigens (representative pictures in Figure 9C and graph in Figure 9D). This confirms the hypothesis produced by single counts of immunogold particles that the amount of autophagoproteasomes are reduced by METH administration.

Merging of LC3+LAMP1 (representative pictures in Figure 9E and graph in Figure 9F) indicates a severe loss of autophagolysosomes. This is likely to be due to an impaired merging between autophagosomes and lysosomes, and this is also compatible with a decrease in chaperone-mediated autophagy. Both conditions are already postulated in the course of degeneration of catecholamine neurons [26,27,28,29,30,31,32,33,34,35,36,37] and METH-induced cytopathology [12].

Merging of LC3+Cat-D (representative pictures in Figure 9G and graph in Figure 9H) is consistent with previous findings indicating a loss of autophagolysosomes. These data are likely to be due to an impaired merging of autophagosomes with lysosomes.

## 3. Discussion

In the present manuscript we provided further evidence about the neuropathology induced by METH on catecholamine cells. In detail, light microscopy provides a measurement of moderate cell loss and consistent cell degeneration induced by METH (100 µM) as assessed following H&E histochemistry and FJ-B fluorescence. The moderate cell death makes it possible to investigate subcellular changes detected at immunohistochemistry and mostly detailed by plain TEM and TEM immunogold. On light microscopy, increased amounts of several antigens involved in cell clearing pathways were documented according to both pioneering and novel studies [9,14,38]. In particular, METH increases LC3, p62, LAMP1, Cat-D, P20S and Mitogreen in the whole cytosol along with the area of overlapping immunohistochemistry signals between each antigen with LC3. The largest overlap was observed for p62, followed by LAMP1, Cat-D, P20S and Mitogreen, respectively. This indicates a quite generalized effect of METH in promoting the expression of some key clearing-pathway-related molecules. These findings were routinely interpreted as the result of increased size of autophagy-related and lysosome-related structures induced by METH. The increase in Mitogreen and the augmented overlap between Mitogreen with LC3 may be the effects of an increased number of damaged mitochondria within autophagolysosomes, the placement of LC3 within mitochondria or both phenomena. When observed by quantitative stoichiometry in situ through immunogold staining, the routine interpretation fades away, since both LC3 and each merging protein co-localize outside the vesicular compartment. In fact, when calculating the ratio between vacuolar and cytosolic amount, each antigen was dissipated from specific vacuoles following METH administration. This point calls for a revision of the significance of increased co-localization of autophagy-related molecules during METH. In particular, the overlapping areas seem to occur outside of autophagolysosomes. This effect occurs concomitantly with a loss of competence of the autophagy machinery, which is described following METH administration [17,39,40,41,42,43,44,45,46,47]. In fact, abundant clusters of tubulo-vesicular structures feature an alteration of specific organelles compared with control cells. In detail, while autophagosomes increase, lysosomes decrease. These data are consistent with a further decrease in the merging between these organelles due to a relented autophagy flux both in parkinsonism [48,49,50,51,52,53,54,55,56] and METH toxicity [47,57,58,59]. Thus, although METH produces the occurrence of a great amount of tubulo-vesicular areas, surpassing two times those measured in controls, the nature of these vesicles is less defined. As reported, following METH the occurrence of these vesicular areas, apart from being more abundant than controls follows a pattern of clustering rather than being homogeneously scattered in the cytosol as observed in controls. The average area of clusters is roughly 2 µm^2^, which corresponds to the cytopathology domains recently described by Ferrucci et al. [12]. However, an uneven distribution of gold particles may occur following immunogold staining, which poses a limit to the study. It is interesting to note that clustering of immunogold is related to treatment rather than unevenly distributed, which provides strength to the present data. Although some concerns exist claiming antigen quantification to be inaccurate following immunogold the present data seem to confirm a quite accurate outcome. It may be claimed that that the density of gold particles does not always directly reflect the amount of antigen present, which is very likely. Nonetheless, it is likely that the ratio between immunogold-bound and non-bound antigen remains the same, which leaves the significance of the results intact ruling out potential misinterpretation beyond unavoidable technical limitations. The present findings provide a specific identification of vesicles occurring within these tubulo-vesicular domains. In fact, METH, apart from increasing these vesicular compartments, alters the relative amount of organelles building up these domains. The occurrence of autophagosomes increase, as well as altered mitochondria, while autophagolysosomes decrease, as well as healthy mitochondria. This is consistent with an engulfment of early autophagy-related structures with an impaired merging between autophagosomes and lysosomes. This relented progression in the autophagy machinery is consistent with the fine mechanisms of disease affecting catecholamine neurons as shown in PD [60,61,62,63,64,65,66,67] and it is consistent with the neuropathology occurring following autophagy inhibition [10]. Thus, it is not surprising that a relented mitophagy generates an increase in altered mitochondria These findings are similar between METH-treated cells [16,68,69,70,71,72,73] and catecholamine neurons during parkinsonism [11,74,75,76,77,78,79,80,81,82,83] and they can be reproduced by administering autophagy inhibitors [10]. This explains why in the present study a great amount of vesicles area consist of mitochondria, most of which are altered compared with controls. Again, a greater number of tubulo-vesicular structures within METH-treated cells were made up of vesicles which could not be reliably identified even when using immunogold for specific antigens. This is likely to depend on a loss of hallmark proteins from appropriate vesicular compartments, which is induced by METH administration. In fact, hallmark proteins were often increased in the whole cells and within non-compartmentalized cytosol following METH, although their compartmentalization within their respective vacuoles was decreased at large. This is consistent with a failure of competence of autophagolysosomes, which is already described following METH [70,73,84,85,86,87,88,89,90], as well as in the course of PD [91,92,93,94,95,96,97] and can be reproduced by autophagy inhibition [10]. In fact, it is noteworthy that most if not all cases of monogenic parkinsonism are produced by mutation in genes involved in this pathway [1,98]. Thus, dissipation of key proteins from autophagolysosome vesicles characterizes METH toxicity and catecholamine degeneration. This subcellular pathology induced by METH is reminiscent of those pathological conditions affecting the central nervous system where increased entropy takes place. In fact, the energy failure induced by METH is expected to produce a decrease in the amount of compartmentalization which governs the appropriate energy-dependent cell metabolism and cell clearance. In fact, very similar data can be documented following ischemia within area penumbra, where most of these antigens lose their appropriate site to act effectively [21,22]. A loss of compartmentalization was documented here also for the remarkable merging of proteasome within autophagy vacuoles as described by Lenzi [99] and Cohen Kaplan [23,24]. This is relevant, since it is postulated that the allocation of P20S within autophagosomes occurs to allow a compartmentalized digestion by proteasome of polyubiquitinated substrates [23,24,99]. This placement of the proteasome is shuttled via p62. In fact, when a dominant-negative variant of p62 is produced, which does not possess the autophagosome binding site known as ubiquitin-associated (UBA) domain, a loss of placement of P20S within autophagosome is described [23,24]. A similar effect is produced by METH, which impedes the placement and activity of P20S within autophagosomes [9,42]. This phenomenon is consistent with the dissipation of p62 outside of autophagy vacuoles here described following METH. Thus, it is not surprising that an increased merging between these proteins takes place following METH, although the site of merging is outside of the vesicular compartment, being placed in surrounding cytosol.

In this way, the effects of METH are reminiscent of the cell pathology, which takes place in PD. In fact, both conditions may be regarded as an autophagolysosomal disorder [62,72,100,101,102,103,104,105,106,107,108,109,110], with impairment of competence within autophagolysosomes, reduced amount and progression through effective cell clearance. This is seminal in generating the accumulation of incompetent vesicular structures. Thus, giant spots of LC3 appear in the cytosol although they do not correspond to giant autophagosomes as previously interpreted. Indeed, these correspond to extra-vacuolar cytosolic domains. In the present study we extended this evidence from LC3 to other proteins, such as p62, P20S, LAMP1 and Cat-D.

In summary, a defect in the autophagolysosome machinery occurs in parkinsonism as well as other degenerative disorders [111,112,113,114,115,116,117], multiple system atrophy [118] and frailty related to aging [119,120,121]. All these conditions provide evidence for a strict overlapping between cytopathology induced by drugs of abuse and neurodegenerative disorders. This manuscript represents a specific addition to understand commonalities between the conditions concerning both protein accumulation, protein dissipation and vesicles packing. In line with these findings it would be interesting to assess whether following METH administration an increased amount of neuromelanin can be detected within catecholamine containing neurons. Such an issue might also be relevant following ex vivo studies where METH is administered chronically, for prolonged time intervals.

## 4. Materials and Methods

### 4.1. Cell Cultures

The PC12 cell line was purchased from a cell bank (IRCCS San Martino Institute, Genova, Italy). Cells were grown in RPMI 1640 medium (Sigma-Aldrich, St. Louis, MO, USA), which was supplemented with heat-inactivated 10% horse serum (HS, Sigma), 5% fetal bovine serum (FBS, Sigma) and penicillin (50 IU/mL)/streptomycin (50 mg/mL, Sigma). The culture was kept under standard conditions in a humidified atmosphere containing 5% CO_2_ at 37 °C. Experiments were carried out during the log-phase of cell growth, when the cells reached approximately 70% confluence [122,123].

For light microscopy experiments, 5 × 10^4^ PC12 cells were seeded on poly-lysine coverslips and placed in 24-well plates with 1 mL/well of culture medium.

For electron microscopy experiments, 1 × 10^6^ PC12 cells were seeded in 6-well plates with 2 mL/well of culture medium.

### 4.2. Hematoxylin and Eosin (H&E) Histochemistry

After removing the medium, cells were washed in PBS and fixed in a 4% paraformaldehyde phosphate buffered solution (PBS) for 15 min, washed in PBS and then immersed for a few minutes in the hematoxylin solution (Sigma-Aldrich). After stopping the hematoxylin staining through repeated washing, cells were plunged for a few seconds within the eosin solution (Sigma-Aldrich) and washed out again to remove the excess of dye. Cells were dehydrated in increasing alcohol solutions, clarified in xylene and finally transferred to a slide to be covered with DPX mounting medium (Sigma-Aldrich) and observed at a Nikon Eclipse Ni light microscope (Nikon, Tokyo, Japan).

### 4.3. FluoroJade-B (FJ-B) Histofluorescence

At the end of the treatment, the medium was removed, cells were washed in PBS and fixed in a solution containing 4% paraformaldehyde in PBS for 5 min. After washing in PBS, cells were incubated in a 0.06% potassium permanganate solution for 10 min at room temperature. Then cells were washed in distilled water and incubated for 20 min in a 0.0004% FJ-B solution obtained from a 0.01% FJ-B stock solution (Merck Millipore, Billerica, MA, USA) diluted in distilled water containing 0.1% acetic acid. After repeated short washing, cells were cover-slipped with DPX mounting medium (Sigma-Aldrich) and were analyzed using a Nikon Eclipse Ni light microscope, equipped with a florescence lamp (Nikon, Tokyo, Japan).

### 4.4. Immunocytochemistry by Light Microscopy

The medium was removed and cells were washed in PBS and fixed with 4% paraformaldehyde in PBS for 15 min, followed by 0.1% Triton X-100 (Sigma-Aldrich) for 15 min in PBS and 10% normal goat serum in PBS for 1 h at room temperature to saturate the aspecific binding sites. Cells were then incubated overnight at 4 °C in 1% normal goat serum in PBS containing the anti-LC3 (Abcam, Cambridge, UK) primary antibody in combination with the following primary antibodies: (i) anti-p62 (Millipore, Burlington, MA, USA) antibody; (ii) anti-LAMP1 (Abcam) antibody; (iii) anti-Cat-D (Abcam) antibody. All primary antibodies were diluted 1:100. After washing in PBS, cells were incubated for 1 h with the appropriate fluorophore-conjugated secondary antibodies, used at a 1:200 dilution, consisting of Alexa488 and Alexa546 (Life Technologies, Carlsbad, CA, USA). The fluorescent dye DAPI (Sigma-Aldrich) was used to stain cell nuclei. Then, cells were washed in PBS, gently transferred to a slide to be finally covered with the mounting medium Fluoroshield (Sigma-Aldrich). Slides were observed using a Nikon Eclipse Ni light microscope, which was equipped with a fluorescent lamp and a digital camera connected to NIS Elements Software (NIS-Elements D 5.30.00 (build 1531) 64-bit) for image analysis (Nikon, Tokyo, Japan). Control sections were incubated with secondary antibodies only.

### 4.5. Transmission Electron Microscopy (TEM)

For the TEM analysis, PC12 cells were centrifuged at 1000× *g* for 5 min, after which they were rinsed in PBS and fixed in paraformaldehyde 2.0% and glutaraldehyde 0.1% for 90 min at pH 7.4 using a 4 °C 0.1 M PBS solution. Following multiple PBS washing, samples were exposed to 1% osmium tetroxide (OsO_4_) for 1 h at 4 °C. Then, they were de-hydrated by increasing concentration of ethanol solutions (from 30%, up to 100%). Samples were finally embedded in epoxy resin. This procedure follows up previous investigations aimed to prepare the samples for both plain and immunogold-based TEM [124]. The use of moderate concentrations of two aldehydes in combination with OsO_4_, and epoxy resin is intended to reduce as much as possible the binding sites of antigens (to be stained with immunogold) while still producing a remarkable contrast distinguish cell organelles and fine intracellular trim (to count organelle area and perform in situ analysis of various cell domains).

Both plain and immunogold-based electron microscopy were carried out on 90 nm thick ultra-thin slices collected on nickel grids.

### 4.6. Plain Electron Microscopy

For plain electron microscopy ultrathin slices were stained with uranyl acetate (to increase the staining of nucleic and amino acids) and lead citrate (to improve the signal provided by heavy metals). In the case of plain electron microscopy samples were observed by using a JEOL JEM SX100 electron-microscope (JEOL, Tokyo, Japan).

### 4.7. Immunogold Electron Microscopy

When antigen binding was needed to count stoichiometry amount of specific proteins, ultrathin slices were placed on aqueous sodium metaperiodate droplets, 30 min, at 22 °C to prevent potential excess of OsO_4_ covering antigen epitopes. Residual sodium metaperiodate was washed out by PBS. Grids were further placed, for 20 min, at 22 °C on droplets of a PBS 1 M blocking solution to prevent non-specific staining. Components of the blocking solution (10% goat serum, 0.2% saponin). Grids were also layered at 4 °C, overnight on drops in which one or two primary antibodies were dissolved. Thus, drops contain antibodies directed against the following antigens: LC3 1:100; p62, 1:100; LAMP1 1:100; P20S 1:100; Cat-D 1:100. All primary antibodies were purchased from Abcam. Drops contained the antibody dissolved in ice-cold PBS solution with 1% goat serum and 0.2% saponin to increase antigen binding. The following day ultrathin sections were layered from grids following were repeated washing in cold PBS (three times, 5 min each). These grids were placed on drops of a blocking buffer (1% goat serum and 0.2% saponin) PBS solution, where gold-conjugated (10 nm or 20 nm immunogold particles) secondary antibodies (diluted 1:100) were dissolved. Gold-conjugated secondary antibodies were provided by BB International, Cardiff, UK). Drops containing 1 or 2 secondary antibody were exposed for 1 h, at 22 °C to measure and compare the amount and co-localization of specific antigens such as LC3 with p62, LC3 with Cat-D, LC3 with LAMP1, LC3 with P20S. Immunogold conjugated antibodies were combined with samples on grids through the exposure to a solution of 1% glutaraldehyde on drops, for 3 min and further washed in distilled water to be further processed as usual for plain electron microscopy, including staining with uranyl acetate and lead citrate. Sliced ultrathin sections were finally observed on TEM (JEOL JEM SX100). Control ultrathin sections were incubated with secondary antibodies only. Each immunogold particles stoichiometry relates to each molecule of antigen (LC3, p62, Cat-D, LAMP1 and P20S).

Immunogold particles where counted to detect in combination various antigens stained either with 10 nm and/or 20 nm immunogold. Counts on TEM were carried out by using a magnification of (8000×), which allows immunogold detection within specific subcellular compartments to assess the stoichiometry amount of each antigen in situ. Counts were expressed considering antigen compartmentalization/dissipation by considering the placement within specific organelles and the widespread occurrence within cytosol. The authentic number of each protein was expressed as the mean ± S.E.M. from n = 30 cells per group.

Comparisons between groups were carried out by using ANOVA with Scheffe’s post hoc analysis; the null hypothesis H_0_ was rejected for *p* < 0.05.

### 4.8. Extended Statistical Analysis (Procedures, Sampling, Bias Inference)

To provide an internal validation of METH-induced cell damage two different procedures were used. In detail, classic H&E staining was combined with a typical marker of neurodegeneration (FJ-B). Remarkably, both markers provided comparable results following administration of METH 100 µM. Thus, based on these previous studies [12,15] a dose–response was not carried out and the dose of METH was selected to produce cytopathology within spared cells. The statistical power of combining two procedures to detect cytopathology at histochemical analysis provides an internal validation of the constancy of these experimental conditions to investigate subcellular alterations within autophagolysosome compartment induced by METH. In addition, this study further validates the efficacy of FJ-B routinely used to assess neurodegeneration as a reliable method to assess METH-induced catecholamine cell toxicity in vitro [12,15]. The amount of cell death corresponds to 37% when assessed with H&E, where the cell death was detected as a lack of cell structures and severe alterations of cell shape and size and faint cytosol staining or dense nuclear staining. In the case of FJ-B, cell damage roughly corresponds to the occurrence of fluorescent cells, which increases significantly (twelvefold compared to controls).

The remarkable effects measured by FJ-B may be due to a higher sensitivity or the occurrence of specific molecules, which produce a fluorescent signal even when present in low amounts. In fact, FJ-B is supposed to provide fluorescent staining for degenerating cells based on the binding of the fluorescent dye to specific polyamine, which increase early during degeneration [125]. This mechanism of action may also lead to some bias since both false positive and false negative may be generated based on the mechanisms of action of METH, which may either increase the amount of polyamines or occlude their presence independently of neurotoxicity. The relevance of METH for the expression of markers stained by FJ-B is presently unknown, which pose a question mark on data interpretation. Nonetheless, adding H&E and assessing the consistency of data between these procedures provides an internal validation on the accuracy of FJ-B to detect in vitro METH-induced cell degeneration. In descriptive statistics for H&E histochemistry viable and non-viable cells were counted under light microscopy, using a 20× magnification; viable H&E-stained cells in each experimental group were counted and they were expressed as mean percentage ± S.E.M. of control (corresponding to 100%). For each treatment group data are expressed as the mean of three separate counts in different though equivalent chambers. When considering viable H&E-stained cells an important issue needs to be clarified since abnormal size, aberrant shape and pathological staining ruled out cell viability [12]. For FJ-B the counts report the mean number ± S.E.M. of degenerating cells (fluorescent cells) for each experimental group, from of three separate experiments assessed under fluorescent microscopy at 20× magnification. Comparisons were calculated by using one-way ANOVA with Scheffe’s post hoc analysis. The null hypothesis was rejected for *p* < 0.05.

When specific antigens were stained in single and double immunostaining, the occurrence fluorescent areas was calculated. In detail, the fluorescent areas of merging was calculated. This was carried out by keeping constant LC3 as a marker of the autophagolysosome compartment. This was variously combined either with p62 or P20S, or LAMP1, or Cat-D or Mitogreen. These double staining provides rough information about: (i) the shuttling of substrates to autophagosomes (p62); (ii) the occurrence of proteasome within autophagosomes (P20S); (iii) the occurrence of mitochondria within autophagosomes (mitophagy, MTG); (iv) the merging of autophagosomes with lysosomes (LAMP1); (v) the merging of autophagosomes with enzyme-containing lysosomes (Cat-D). Areas of merging were expressed either as the mean ± S.E.M. from 90 control cells and 90 METH-treated cells or they were expressed as the mean percentage ±S.E.M. of the merging areas measured in controls (arbitrarily assumed as 100%). Comparisons were calculated by using one-way ANOVA with Scheffe’s post hoc analysis. The null hypothesis was rejected for *p* < 0.05.

For electron microscopy cytosol of control and METH-treated cells was sampled and the percentage of tubulo-vesicular areas was measured. These roughly corresponds to cytosolic domains owing an area of approximately 2 µm^2^, which in turn corresponds to METH-induced pathological spots as previously shown [12]. In fact, in control cells the distribution of tubulo-vesicular structures was quite uniform in the cytosol, while following METH these areas increased and clustered within specific spots owing an area of roughly 2 µm^2^. Therefore, to carry out uniform counts, only those unusual domains from controls owing a tubulo-vesicular area of 2 µm^2^ wide were considered. These domains were much more abundant (4.5-fold) in METH-treated cells. For inferential statistics we selected 30 tubulo-vesicular areas from 30 controls (which required a number or roughly 120 cells to be harvested) and 30 tubulo-vesicular areas from METH-treated cells (which required a number or roughly 30 cells to be harvested). These areas were analyzed concerning the composition of each specific recognizable organelle and data for each organelle are expressed as the mean percentage ±S.E.M. considering the total mean vesicular area in the domain of 2 µm^2^ as 100%. Comparisons were calculated by using one-way ANOVA with Scheffe’s post hoc analysis. The null hypothesis was rejected for *p* < 0.05. When occurrence of specific hallmark proteins was assessed in situ by quantitative stoichiometry, immunogold was used as a reference (Bergensen et al., 2000 [11]) and the amount of immunogold particles binding with a 1:1 ratio to hallmark antigens provided a quantitative measurement of each protein in situ. In detail, LC3 particles were counted within tubulo-vesicular compartments (aiming at cytosolic vacuolar structures as reported in representative figures) along with the number of mitochondria or their remnants, the amount of p62, P20S, LAMP1 and Cat-D. Each antigen was counted alone or in combination with LC3.

In detail, the site-specificity of in situ stoichiometry allowed to measure the real amount of each protein in each specific compartment [9,16,126]. This allow to count the variation of specifically stained vacuoles under the effects of METH compared with controls. Again, the amount of each protein within the whole cytosol and within specific vacuoles was measured and the effects of METH were calculated measuring either the increase or decrease in the protein within cytosol and within vacuoles. This allows to infer how the compartmentalization of the protein was modified under the effects of METH. This was expressed by comparing the ratio of vacuolar protein/cytosol protein. Each value was expressed as the mean ±S.E.M. of 30 cells per group. Comparisons were calculated by using one-way ANOVA with Scheffe’s post hoc analysis. The null hypothesis was rejected for *p* < 0.05.

## Figures and Tables

**Table 1:** Overlapping area (µm^2^) between each antigen and LC3 observed on light microscopy in controls and after METH treatment.

	CONTROL	METH
**LC3 + p62**	1.02 µm^2^ ± 0.13 µm^2^	7.8 µm^2^ ± 0.6 µm^2^ *
**LC3 + P20S**	0.97 µm^2^ ± 0.06 µm^2^	4.4 µm^2^ ± 0.2 µm^2^ *
**LC3 + LAMP1**	1.0 µm^2^ ± 0.05 µm^2^	6.1 µm^2^ ± 0.3 µm^2^ *
**LC3 + Cat-D**	0.88 µm^2^ ± 0.1 µm^2^	4.3 µm^2^ ± 0.2 µm^2^ *
**LC3 + Mitogreen**	0.87 µm^2^ ± 0.07 µm^2^	3.8 µm^2^ ± 0.2 µm^2^ *

* *p* < 0.05 compared with controls.

**Table 2:** Transmission electron microscopy reveals the abundant tubulo-vesicular structures following METH in PC12 cells.

	CONTROLS	METH
**Autophagosomes**	22.34 ± 1.32%	27.08 ± 1,05% *
**Lysosomes**	18.65 ± 1.10%	4.19 ± 0.37% *
**Damaged mitochondria**	6.40 ± 0.50%	14.21 ± 1.00% *
**Healthy mitochondria**	10.87 ± 0.49%	4.07 ± 0.28% *
**Multivesicular bodies (MVBs)**	5.36 ± 0.23%	5.02 ± 0.30%
**Others**	36.38 ± 1.80%	45.43 ± 1.69% *

* *p* < 0.05 compared with controls.

## Data Availability

The data that support the findings of this study will be made available on request through a repository file.
